# Bacteria Regulate Intestinal Epithelial Cell Differentiation Factors Both *In Vitro* and *In Vivo*

**DOI:** 10.1371/journal.pone.0055620

**Published:** 2013-02-13

**Authors:** Svetlana Becker, Tobias A. Oelschlaeger, Andy Wullaert, Manolis Pasparakis, Jan Wehkamp, Eduard F. Stange, Michael Gersemann

**Affiliations:** 1 Dr. Margarete Fischer-Bosch Institute for Clinical Pharmacology, Stuttgart, Germany; 2 University of Tübingen, Tübingen, Germany; 3 Institute for Molecular Biology of Infection, University of Würzburg, Würzburg, Germany; 4 Institute for Genetics, University of Cologne, Cologne, Germany; 5 EMBL Mouse Biology Unit, Monterotondo, Italy; 6 Department of Internal Medicine I, Robert Bosch Hospital, Stuttgart, Germany; University of Auvergne, France

## Abstract

**Background:**

The human colon harbours a plethora of bacteria known to broadly impact on mucosal metabolism and function and thought to be involved in inflammatory bowel disease pathogenesis and colon cancer development. In this report, we investigated the effect of colonic bacteria on epithelial cell differentiation factors *in vitro* and *in vivo*. As key transcription factors we focused on Hes1, known to direct towards an absorptive cell fate, Hath1 and KLF4, which govern goblet cell.

**Methods:**

Expression of the transcription factors Hes1, Hath1 and KLF4, the mucins Muc1 and Muc2 and the defensin HBD2 were measured by real-time PCR in LS174T cells following incubation with several heat-inactivated *E. coli* strains, including the probiotic *E. coli* Nissle 1917+/− flagellin, *Lactobacilli* and *Bifidobacteria*. For protein detection Western blot experiments and chamber-slide immunostaining were performed. Finally, mRNA and protein expression of these factors was evaluated in the colon of germfree vs. specific pathogen free vs. conventionalized mice and colonic goblet cells were counted.

**Results:**

Expression of Hes1 and Hath1, and to a minor degree also of KLF4, was reduced by *E. coli* K-12 and *E. coli* Nissle 1917. In contrast, Muc1 and HBD2 expression were significantly enhanced, independent of the Notch signalling pathway. Probiotic *E. coli* Nissle 1917 regulated Hes1, Hath1, Muc1 and HBD2 through flagellin. *In vivo* experiments confirmed the observed *in vitro* effects of bacteria by a diminished colonic expression of Hath1 and KLF4 in specific pathogen free and conventionalized mice as compared to germ free mice whereas the number of goblet cells was unchanged in these mice.

**Conclusions:**

Intestinal bacteria influence the intestinal epithelial differentiation factors Hes1, Hath1 and KLF4, as well as Muc1 and HBD2, *in vitro* and *in vivo*. The induction of Muc1 and HBD2 seems to be triggered directly by bacteria and not by Notch.

## Introduction

The colon provides the most favorable conditions for intestinal microbiota and harbors, with approximately 10^12^ microorganisms per gram of intestinal content, the most densely populated and complex community of the human gastrointestinal tract [1], [2]. During evolution a complex and intensive mutualistic relationship between bacteria and host has developed. The intestinal microflora influences the host in different ways by modulating the immune system, protecting against pathogen invasion and attachment, and contributing to digestion and nutritional uptake [3].

In a healthy gut the synergistic co-existence of intestinal microflora and the host is secured by an intact mucosal barrier. The barrier is provided by the intestinal epithelium, consisting of absorptive, goblet, Paneth and neuroendocrine cells, separating the intestinal wall from the luminal microbes. Goblet cells secrete mucins, e.g. Muc1 and Muc2 as structural proteins of the protective mucus layer covering the whole gastrointestinal tract [4]. Moreover, epithelial cells produce broad-spectrum antimicrobial peptides, including defensins [5]–[8]. Once secreted, the small cationic defensins are fixed in the negatively charged mucus [9]. This mucus barrier is the first front of gut defence shielding the intestinal wall from luminal microbiota.

The intestinal epithelium differentiates from multipotent stem cells located at the bottom of the crypt and undergoes a rapid and continuous regeneration [10]. This process is regulated by a complex network of different differentiation signals. For example, the early determination of secretory versus absorptive cells is regulated by antagonistic interplay of the Notch target gene Hes1 and the basic helix–loop–helix transcription factor Hath1. In progenitor cells expressing Hes1, Hath1 gene expression is blocked, directing the cells to the absorptive fate. In contrast, in progenitors with inactive Notch/Hes1 signaling, the Hath1 gene can be transcriptionally activated and these cells transit to the secretory lineage [11], [12]. The predetermined cells of the secretory line require additional signals for differentiation to specific cell types such as, for goblet cells, the zinc-finger transcription factor KLF4 [13].

Dysregulation of this regulatory network may lead to defective epithelial differentiation and finally to altered function of the mucosal barrier as shown in both forms of inflammatory bowel diseases (IBD), Crohn’s disease (CD) and ulcerative colitis (UC) [14], [15]. There is mounting evidence that the commensal intestinal microbiota plays a key role in the pathogenesis of IBD [16]. Among other evidence, this is underlined by the observation that patients with IBD have more mucosa-adherent bacteria, some of which are even found intracellularly [17], [18]. Moreover, recent studies linked intestinal epithelial differentiation to IBD development. For instance, ileal CD is associated with defective Wnt mediated Paneth cell differentiation and consequently with a diminished production of the defensins HD5 and HD6 resulting in decreased mucosal antibacterial activity [19], [20]. In active UC, defective Hath1 expression [21], [22] is associated with a decreased number of mature goblet cells in the upper part of the colonic crypt [21]. As a consequence, mucin synthesis in active UC is defective leading to a diminished mucus layer [23], [24]. In both cases, these epithelial differentiation defects may lead to invasion of the luminal bacteria into the mucosa where they could trigger inflammation. Additionally, there is evidence that bacteria could contribute to colon cancer development. For instance, animal models showed carcinogenic properties in some bacterial species [25], [26]. Moreover, patients with colorectal cancer exhibit bacteria adhering to tumor tissue [27] and have indirect evidence of bacterial invasion [28], [29].

The aim of the present study was to elucidate whether and how bacteria regulate intestinal epithelial cell differentiation. The effects of microbiota on the expression of epithelial differentiation factors Hath1, KLF4 and Hes1, as well as the mucins Muc1, Muc2 and the defensin HBD2 were analysed *in vitro* and *in vivo.* This way, we aimed to get more insight into the complex interplay between bacteria and differentiation with regard to the potential impact of the microbiota on intestinal inflammation and cancer development.

## Materials and Methods

### Cell Culture Experiments

The colon adenocarcinoma cell line LS174T (American Type Culture Collection, Manassas, USA) was cultivated in Dulbecco’s modified Eagle medium (DMEM, Gibco Life Technologies, Eggenstein, Germany) in a humidified atmosphere at 37°C and 5% CO_2_. 10% fetal calf serum (FCS, PAA Laboratories, Pasching, Austria), 1% non-essential amino acids (Gibco Life Technologies), 1% penicillin/streptomycin (Gibco Life Technologies) and 1% sodium pyruvate (Gibco Life Technologies) was added. For experiments, cells were seeded in 12-well culture plates (Becton Dickinson, Franklin Lakes, New Jersey, USA) at a density of 0.65×10^6^ per well and grown to about 70% confluence. Then cells were washed with phosphate-buffered saline (PBS, Gibco Life Technologies) and incubated in FCS- and antibiotic-free DMEM for 12 hours.

To investigate the possible role of several bacteria in the regulation of epithelial differentiation, LS174T cells were treated with heat-inactivated *E. coli* strains Symbioflor G1, G2 and G3, *Escherichia coli* K-12, *E. coli* Nissle 1917, *Lactobacillus fermentum* and *acidophilus*, *Bifidobacterium longum*, *breve* and *adolescentis* as well as *Bacteroides vulgatus* for 3 and 12 hours. LS174T cells were also incubated for 3 hours with *E. coli* Nissle 1917 wild type and *E. coli* Nissle 1917 mutant strains EcNΔ*fliA,* EcNΔ*fliC,* EcNΔ*flgE,* EcNΔ*fim*, EcNΔ*foc* and EcNΔ*csgBA*, which were kindly provided by T. Oelschlaeger (Institute for Molecular Biology of Infection, University of Würzburg, Germany). The characteristics of the used bacterial strains are summarized in table 1. All *E. coli* strains and *B. vulgatus* were grown under aerobic, *Lactobacilli* and *Bifidobacteria* under anaerobic conditions as described previously [30]. For experiments, bacteria were heat-inactivated in a water bath at 65°C for 1 hour, washed with PBS and adjusted to a density of 3×10^8^ cells/ml with FCS- and antibiotic-free DMEM.

**Table 1:** Characteristics of the bacterial strains.

Strain	Stereotype	Characteristics/Isolatetypes/Deletions*	Source
E. coli Nissle 1917 (DSM 6601)	O6:K5:H1	Apathogen, pharmaceutical strain	ACS
EcNΔfliA		Sigma factor of flagella genes*	WÜR
EcNΔfliC		flagellin*	
EcNΔflgE		hook*	
EcNΔfim		Type 1 pili*	
EcNΔfoc		F1C pili*	
EcNΔcsgBA		Curli-negative*	
E. coli K-12	DSM 498	Reference strain	DSMZ
L. fermentum	PZ 1162	Intestinal isolate	ACS
L. acidophilus	PZ 1138	Industrial probiotic strain	ACS (GR)
B. longum (DSM 20219T)	PZ 1323	Intestinal isolate	ACS
B. breve	Ha6/14c	Intestinal isolate	ACS
B. adolescentis TSD	PZ 4009	Intestinal isolate	ACS
B. vulgatus	DSM 1447	Intestinal isolate	DSMZ
E. coli DSM 17252			SYM
S2 G1: E. coli	Osp.:H-	Probiotic strain	
Genotype 1/2			
S2 G2: E. coli	O 13.:H-	Probiotic strain	
Genotype 3/10			
S2 G3: E. coli	Osp.:H-	Probiotic strain	
Genotype 4/10			

EcN…*E. coli* Nissle 1917.

ACS: Ardeypharm collection of strains, Pharma-Zentrale GmbH, Herdecke, Germany.

WÜR: Collection of strains, University of Würzburg.

DSMZ: German Collection of Microorganisms and Cell cultures, Braunschweig, Germany.

GR: Strain collection of G. Reuter (strain deposited by Mitusoka at the Japanese Collection of Microorganisms).

SYM: SymbioPharm GmbH, Germany.

The possible involvement of Notch signalling was investigated by the treatment of LS174T cells with the γ-secretase (Notch) inhibitor dibenzazepine (DBZ, Axon Medchem, Groningen, Netherlands) in a concentration of 1 µM (in 0.1% DMSO in DMEM) for 3, 6, 12 and 24 hours in absence or presence of *E. coli* Nissle 1917.

After incubation, LS174T cells were rinsed in PBS and mRNA (for PCR measurements) or total protein (for Western blot experiments) was isolated as described below. All cell culture experiments were performed for at least 3 independent times in triplicates.

### Mouse Tissue

Colonic samples (mRNA and tissue from whole mouse colonic mucosa) of mice housed in germfree conditions at the University of Ulm, mice housed in specific pathogen free (SPF) conditions at the University of Cologne and germfree mice that were transferred to the SPF facility in Cologne for cohousing with SPF mice for 4 weeks (conventionalized) (all C57Bl/6), were kindly provided by M. Pasparakis (Institute for Genetics, Centre for Molecular Medicine University of Cologne).

All animal procedures were conducted in accordance with European, national and institutional guidelines and protocols and were approved at 06.08.2008 from the ethics committee of Tübingen (Regierungspräsidium AZ 35/9185.81-3), Research-Nr. 929.

### RNA Isolation and Reverse Transcription

Treated LS174T cells were washed with PBS and harvested by scraping. Total RNA from the cell lysates was isolated and DNAse digestion was performed as recommended by the supplier to avoid genomic DNA contamination (RNeasy Mini Kit, Qiagen, Hilden, Germany). Subsequently 1 µg of total RNA was reverse transcribed into cDNA with oligo (dT) primers and 15 U/µg AMV Reverse Transcriptase (Promega, Madison, USA) according to standard procedures. RNA preparations were used for PCR analysis.

### Quantitative Real-time Reverse Transcriptase PCR

For mRNA quantification, real-time PCR was performed in a SYBR Green fluorescence temperature cycler (LightCycler®, Roche Diagnostics, Mannheim, Germany). Single-stranded cDNA (or gene-specific plasmids as controls) corresponding to 10 ng of RNA served as a template for PCR with specific oligonucleotide primer pairs (table 2) as described previously [31]. All primers were checked for specific binding to the sequence of interest using BLAST. Plasmids for each product were synthesized with the TOPO TA Cloning Kit (Invitrogen, Carlsbad, CA, USA) according to the supplier’s protocol. PCR-amplified DNA fragments were confirmed by sequencing. The correctly sequenced plasmids were serially diluted for internal standard curves. The mRNA data were normalized to the mRNA of ß-actin.

**Table 2:** Oligonucleotide primer pairs used for PCR measurements.

Product	Forward primer (5′−>3′)	Reverse primer (5′−>3′)
**ß-actin**	GCCAACCGCGAGAAGATGA	CATCACGATGCCAGTGGTA
**Hath1**	CGAGAGAGCATCCCGTCTAC	TCCGGGGAATGTAGCAAATA
**KLF4**	CCCACACAGGTGAGAAACCT	ATGTGTAAGGCGAGGTGGTC
**Hes1**	CTCTCTTCCCTCCGGACTCT	AGGCGCAATCCAATATGAAC
**Muc1**	AGACGTCAGCGTGAGTGATG	CAGCTGCCCGTAGTTCTTTC
**Muc2**	ACCCGCACTATGTCACCTTC	GGGATCGCAGTGGTAGTTGT
**HBD2**	ATCAGCCATGAGGGTCTTGT	GAGACCACAGGTGCCAATTT
**mß-actin**	GCTGAGAGGGAAATCGTGCGTG	CCAGGGAGGAAGAGGATGCGG
**Math1**	AGAGACCTTCCCGTCTACCC	CTGCAAAGTGGGAGTCAGC
**mHes1**	AGAGGCGAAGGGCAAGAATA	CGGAGGTGCTTCACAGTCAT
**mKLF4**	AGAGGAGCCCAAGCCAAAGAGG	CCACAGCCGTCCCAGTCACAGT
**mMuc1**	GAAGACCCCAGCTCCAACTA	GGAGCCTGACCTGAACTTGA
**mMuc2**	GTGTGGGACCTGACAATGTG	ACAACGAGGTAGGTGCCATC

m … mouse.

### Protein Preparation

LS174T cells were washed with PBS, harvested by scraping and centrifuged for two times at 1300 rpm for 5 minutes. Whole cell lysates were extracted with lysis buffer containing 20 mM Tris-HCl pH 7.5, 150 mM NaCl, 1 mM EDTA, 1% Trition X-100, 25 mM Sodiumpyrophosphat, 1 mM Glycerolphosphat, 1 mM Na_3_VO_4_, 6M Urea and 1% Protease Inhibitor Cocktail (Sigma-Aldrich Chemie GmbH, Steinheim, Germany). Mouse colonic tissue was extracted with lysis buffer (see above) and homogenized by FastPrep instrument. Mouse and cell culture lysates were centrifuged at 13000 rpm at 4°C for 20 minutes and the supernatant was collected. Total protein amount was measured with the Bicinchoninic Acid Protein Assay (Smith) as described previously [32]. Isolated proteins were used for Western blot experiments.

### Western Blot

40 µg (LS174T cell lysates) or 20 µg (mouse colonic tissue) of total protein was separated on a 10% Tris-glycin SDS polyacrylamide gel, transferred to 0.45 µm pore size nitrocellulose membranes (Schleicher & Schuell, Keene, NH, USA) and blocked with 5% skimmed milk powder in TBST (10 mM Tris pH 8.0, 150 mM NaCl, 0.05% Tween 20) for 1 hour. Then, the membranes were washed with TBST and incubated overnight at 4°C with the primary antibodies. Anti-Hath1/Math1 (AB5692, Millipore, Temecula, CA, USA) and anti-Hes1 (sc-25392, Santa Cruz Biotechnology, Heidelberg, Germany) antibodies were diluted 1∶200 in 5% skimmed milk powder in TBST, whereas the dilution of the anti-KLF4 (ab26648, Abcam, Cambridge, USA) antibody was 1∶100. After repeatedly washing, the membranes were treated for 1 hour with the secondary HRP-conjugated goat anti-rabbit immunoglobulin G antibody (Immuno Research Laboratories, West Grove, PA, USA; dilution 1∶5000). Then, protein was detected with the Amersham TM ECL Plus Western Blotting Detection System (GE Healthcare, Chalfont St Giles, UK) and signals were visualized with a chemiluminescence camera charge-coupled device LAS-1000 (Fuji, Tokio, Japan). Densitometric analysis was performed with AIDA 2.1 software (Raytest, Straubenhardt, Germany). ß-actin antibody (Sigma-Aldrich) was used as an internal control.

### Immunostaining and Goblet Cell Count

For Muc1 and Muc2 staining, LS174T cells were seeded in 2-well Chamber Slides (Nalge Nunc International Corp., Naperville, IL, USA) at a density of 0.65×10^6^ per well. Cells were incubated with *E. coli* Nissle 1917 for 6 hours, washed with PBS and fixed with 100% ethanol for 10 minutes at −20°C. Then, ethanol was removed and the slides were rinsed for two times with TBST. Immunostaining and visualisation was performed as previously described [33]. Anti-Muc1 antibody (VU4H5, Santa Cruz Biotechnology, Heidelberg, Germany) was diluted 1∶20 and Anti-Muc2 (NCL-Muc2, Leica Biosystems Newcastle Ltd, Balliol Business Park West, United Kingdom) antibody 1∶200 in DAKO REAL Antibody dilution buffer (Dako, Glostrup, Denmark). Cells were also counterstained with hematoxylin.

The number of goblet cells was determined in sections from mouse colonic tissue (germfree: n = 6, SPF housed: n = 4, conventionalized: n = 4) following a standard Alcian Blue staining by blindly counting the Alcian Blue positive vacuoles in a total of 10 crypts per mice.

### Statistics

Quantitative real-time PCR and Western blot results were analysed using the Mann-Whitney test. Values of p<0.05 were considered to be statistically significant. All statistical analyses were performed and all graphs were generated with the GraphPad Prism version 5.0 software. Data are presented as means with standard error of the mean (SEM).

## Results

### Hes1, Hath1 and KLF4 are Regulated by Bacteria *in vitro*

**Figure 1:**
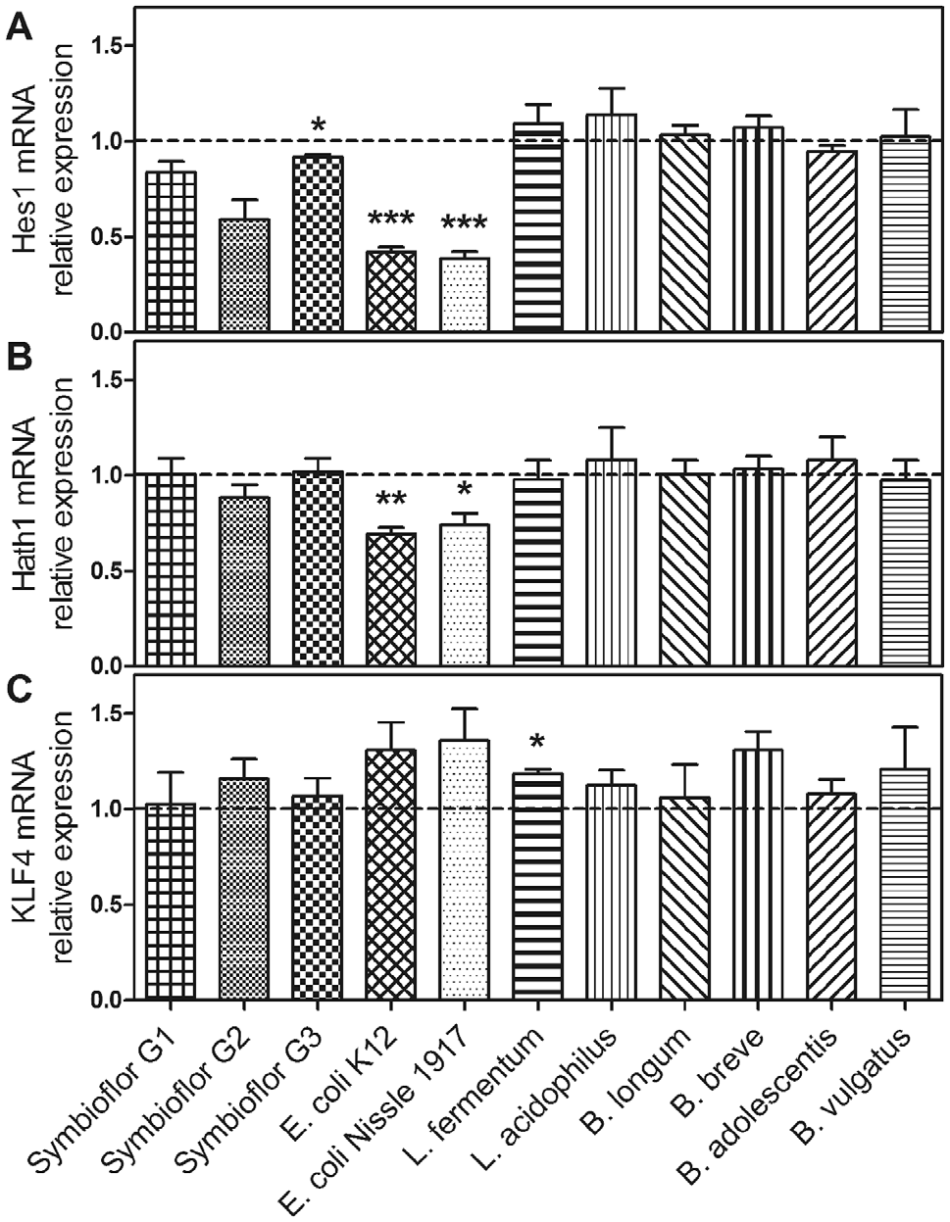
Hes1, Hath1 and KLF4 mRNA expression in LS174T cells after treatment with different heat-inactivated bacteria for 3 hours. Hes1 expression was impaired by Symbioflor G3, *E. coli* K-12 and *E. coli* Nissle 1917 (A). Hath1 transcripts were downregulated by *E. coli* K-12 and *E. coli* Nissle 1917 (B). KLF4 mRNA was augmented by *L. fermentum* (C). Data represent the means ± SEM normalised to basal expression of untreated controls set at 1 (n = 4). *: p<0.05, **: p<0.01, ***: p<0.001. For 12 hours treatment results see Fig. S1.

**Figure 2:**
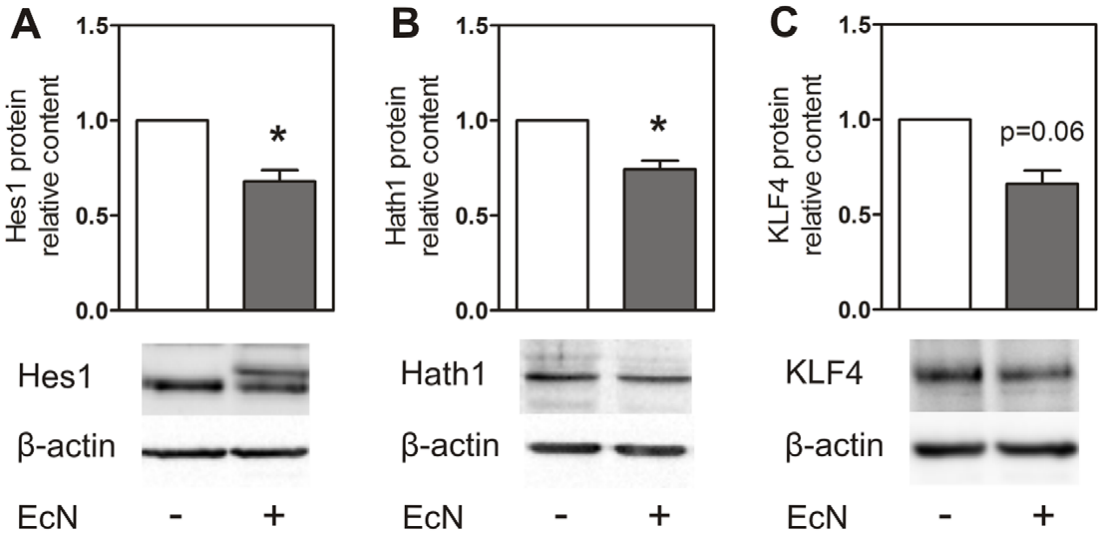
Hes1, Hath1 and KLF4 protein expression (Western blot) in LS174T cells after treatment with heat-inactivated *E. coli* Nissle 1917. Hes1 Western blot analysis showed a double band after incubation (A). The protein content of the lower band (equivalent to the control band) was significantly decreased in comparison to controls after 12 hours of treatment (A). Hath1 protein was significantly decreased after 6 hours treatment with *E. coli* Nissle 1917 (B). KLF4 was clearly downregulated after 24 hours of treatment (C). Data represent the means ± SEM normalised to basal expression of untreated controls set at 1 (n = 3). *: p<0.05.

First, we analysed mRNA expression of the epithelial cell differentiation markers Hes1, Hath1 and KLF4 in LS174T cells following treatment with different heat-inactivated bacteria. Hes1 transcripts (Fig. 1A and Fig. S1A) were diminished following incubation with *E. coli* K-12 (3 hours: 0.42-fold, p<0.001; 12 hours: 0.64-fold, p<0.001) and *E. coli* Nissle 1917 (3 hours: 0.38-fold, p = 0.001; 12 hours: 0.67-fold, p<0.001). Moreover, 3 hours treatment with Symbioflor G3 (0.92-fold, p = 0.023), as well as 12 hours treatment with Symbioflor G2 (0.75-fold, p = 0.043) and *L. acidophilus* (0.79-fold, p = 0.030) also led to a downregulation of Hes1 mRNA. Hes1 Western blot analysis (Fig. 2A) showed an appearance of a higher molecular weight band after treatment with *E. coli* Nissle 1917. However, densitometric analysis of the lower molecular weight band that is also present in control cells revealed a significant reduction in Hes1 protein levels after 12 hours of *E. coli* Nissle 1917 stimulation (0.71-fold, p = 0.047).

Hath1 mRNA levels (Fig. 1B and Fig. S1B) were also significantly downregulated by treatment with *E. coli* K-12 (3 hours: 0.69-fold, p = 0.002; 12 hours: 0.85-fold, p = 0.008) and *E. coli* Nissle 1917 (3 hours: 0.74-fold, p = 0.025; 12 hours: 0.80-fold, p = 0.001). This *E. coli* Nissle 1917 effect on Hath1 mRNA expression was confirmed by Western blot analysis showing Hath1 protein levels to be significantly decreased after 6 hours of bacterial exposure (0.71-fold, p = 0.038, Fig. 2B).

KLF4 mRNA transcripts (Fig. 1C and Fig. S1C) were significantly induced after a 3 hour treatment with *L. fermentum* (1.2-fold, p = 0.011, Fig. 1C) and significantly reduced after 12 hours of treatment with *E. coli* K-12 (0.81-fold, p = 0.005), *E. coli* Nissle 1917 (0.83-fold, p = 0.008), *L. acidophilus* (0.77-fold, p = 0.008) and *B. vulgatus* (0.77-fold, p = 0.003). KLF4 protein levels were also slightly downregulated after 24 hours incubation with *E. coli* Nissle 1917 (0.69-fold, p = 0.06, Fig. 2C).

### HBD2 and Muc1 but not Muc2 are Regulated by Bacteria *in vitro*

Since downregulation of both Hes1 and Hath1 expression levels could lead to differentiation to either the absorptive or the secretory cell lineage, we further investigated the effect of bacteria on epithelial differentiation by analysing the expression of HBD2, Muc1 and Muc2 in LS174T cells after treatment with different bacteria strains.

**Figure 3:**
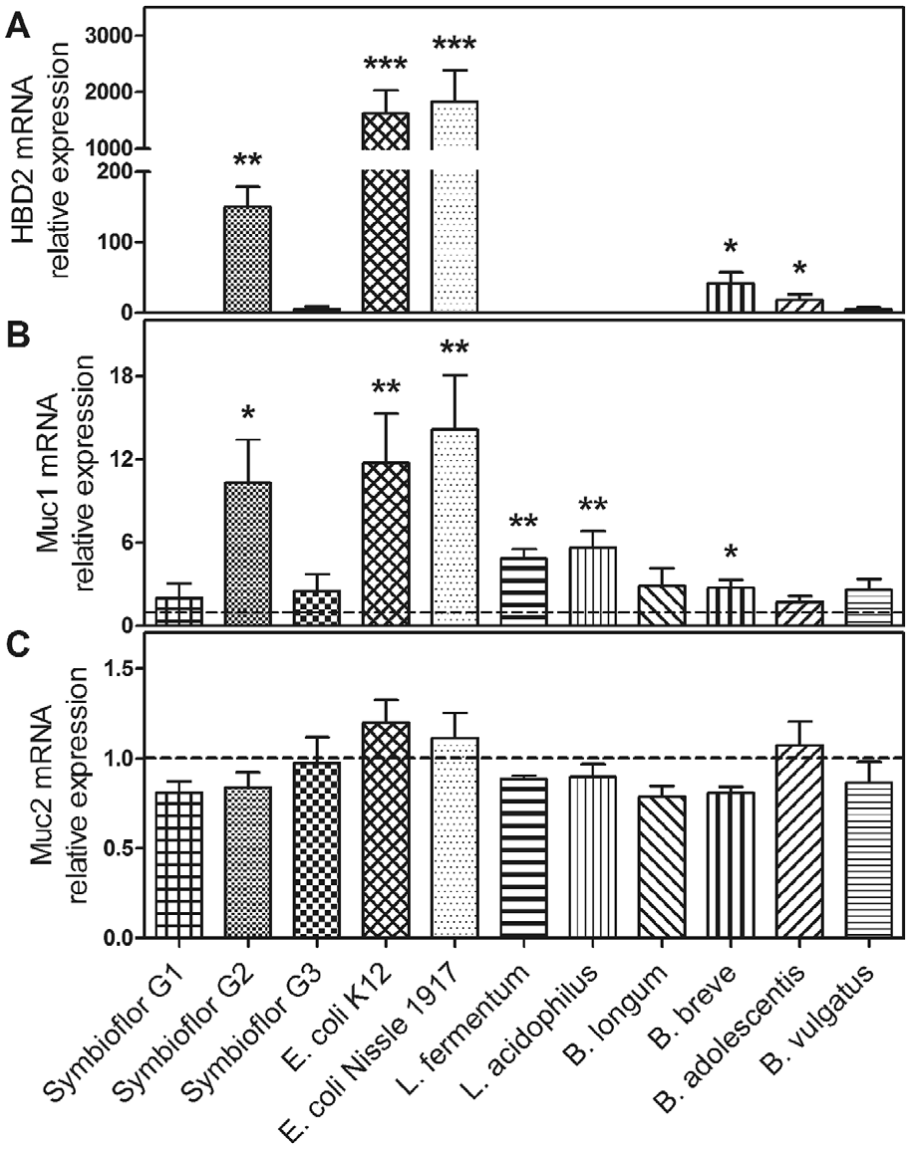
HBD2, Muc1 and Muc2 mRNA expression in LS174T cells after treatment with different heat-inactivated bacteria for 3 hours. HBD2 expression was induced by Symbioflor G2, *E. coli* K-12, *E. coli* Nissle 1917, *B. breve* and *adolescentis* (A). Muc1 transcripts were upregulated by Symbioflor G2, *E. coli* K-12, *E. coli* Nissle 1917, *L. fermentum* and *acidophilus* as well as *B. breve* (B). Muc2 mRNA was unchanged (C). Data represent the means ± SEM normalised to basal expression of untreated controls set at 1 (n = 4). *: p<0.05, **: p<0.01, ***: p<0.001. For 12 hours treatment results see Fig. S2.

HBD2 mRNA (Fig. 3A and Fig. S2A) was induced by Symbioflor G2 (3 hours: 150-fold, p = 0.002; 12 hours: 185-fold, p<0.001), *E. coli* K-12 (3 hours: 1630-fold, p<0.001; 12 hours: 1310-fold, p<0.001), *E. coli* Nissle 1917 (3 hours: 1833-fold, p<0.001; 12 hours: 1578-fold, p<0.001) and *B. breve* (3 hours: 42-fold, p = 0.018; 12 hours: 61-fold, p = 0.005) for both time-points, whereas *B. adolescentis* (18-fold, p = 0.032) led to a significant increase of HBD2 transcripts only after 3 hours of treatment.

**Figure 4:**
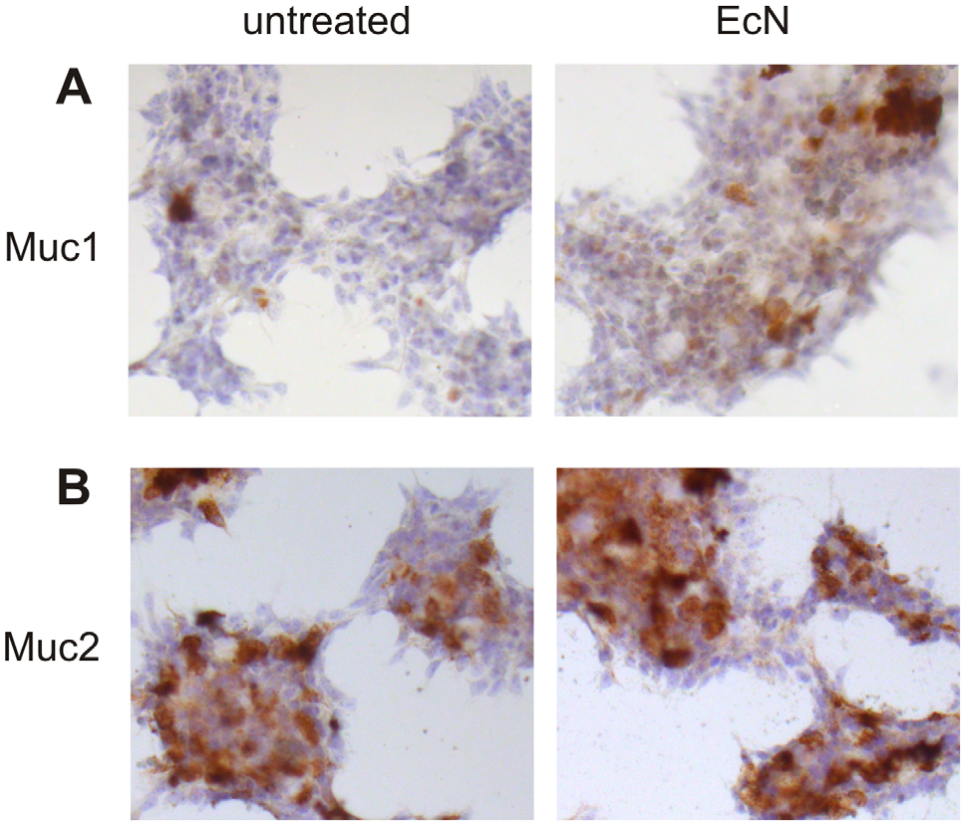
Muc1 and Muc2 protein expression (immunostaining) in LS174T cells after treatment with heat-inactivated *E. coli* Nissle 1917. Staining of Muc1 (A) but not Muc2 (B) was more pronounced following incubation with *E. coli* Nissle 1917 for 6 hours (representative example of 3 stainings).

Muc1 mRNA transcripts (Fig. 3B and Fig. S2B) were significantly augmented following a 3 hour stimulation with Symbioflor G2 (10-fold, p = 0.026), *E. coli* K-12 (12-fold, p = 0.002), *E. coli* Nissle 1917 (14-fold, p = 0.002), *L. fermentum* (4.9-fold, p = 0.002) and *acidophilus* (5.6-fold, p = 0.010), as well as *B. breve* (2.7-fold, p = 0.049). After 12 hours of treatment the Muc1 induction was still significant for Symbioflor G2 (2.9-fold, p = 0.027), *E. coli* K-12 (2.7-fold, p = 0.018) and *E. coli* Nissle 1917 (3.3-fold, p = 0.008). This increase of Muc1 mRNA expression after exposure to *E. coli* Nissle 1917 was confirmed on the protein level by immunocytochemistry (Fig. 4A).

Muc2 mRNA (Fig. 3C and Fig. S2C) expression was unchanged after exposure to intestinal bacteria. Accordingly, incubation with *E. coli* Nissle 1917 had no effect on Muc2 protein content (Fig. 4B).

To clarify whether the effects on HBD2 and Muc1 expression are caused by bacterial treatment or indirectly by changes in Hes1 and Hath1 expression, we blocked the Notch pathway in LS174T cells using the gamma-secretase inhibitor DBZ up to 24 hours with and without *E. coli* Nissle. The DBZ treatment led to a strong downregulation of Hes1 (3 h: 0.34-fold, p = 0.01; 6 h: 0.11-fold, p = 0.0003; 12 h: 0.09-fold, p<0.0001 and 24 h: 0.11-fold, p = 0.001) followed by a delayed Hath1 upregulation (3 h: 0.93-fold, n.s.; 6 h: 1.19-fold, p = 0.0206; 12 h: 2.01-fold, p = 0,0297 and 24 h: 2.44-fold, p = 0.0032), without affecting HBD2, Muc1 or Muc2 expression. Thus, no significant differences in mRNA expression of these products in untreated and DBZ treated cells as well as no differences between *E. coli* Nissle only and *E. coli* Nissle+DBZ treated cells were observed (Fig. S3 and S4). Since HBD2 and Muc1 are induced by *E. coli* Nissle 1917 independently of Notch pathway inhibition, we suggest that this induction is triggered by bacteria or their components and not through changes in Hes1 or Hath1 expression.

### *E. coli* Nissle 1917 Flagellin is Required for Regulating Hes1, Hath1, HBD2 and Muc1 Expression

Since previous data mechanically linked the inducing effect of *E. coli* Nissle 1917 on HBD2 to its flagellin [34], we analyzed whether flagellin is essential to regulate expression of Hes1, Hath1 and Muc1. LS174T cells were incubated for 3 hours with *E. coli* Nissle 1917 wild type strain as well as various *E. coli* Nissle 1917 deletion mutants as listed in table 1.

**Figure 5:**
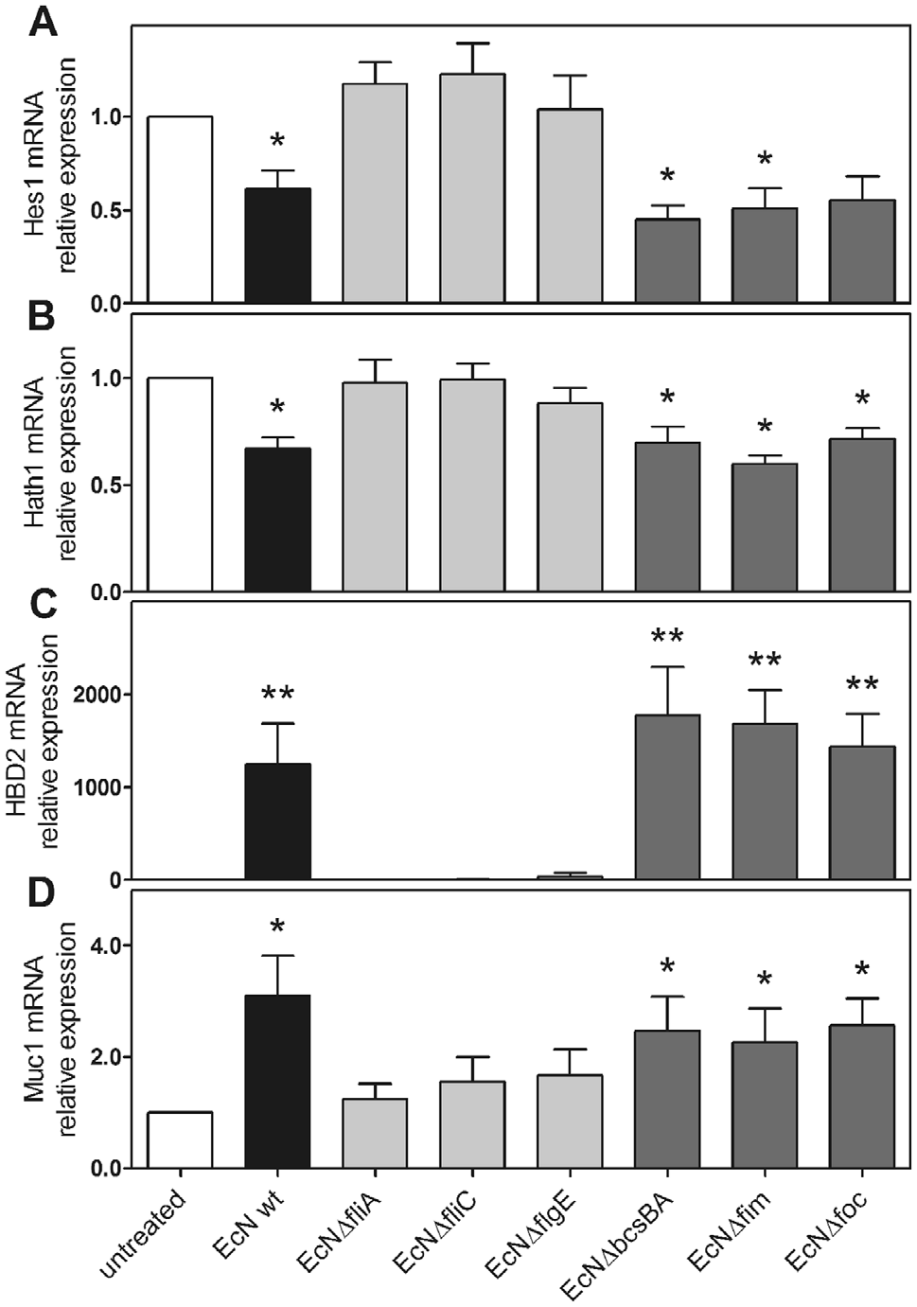
Hes1, Hath1, HBD2 and Muc1 mRNA expression in LS174T cells incubated with heat inactivated *E. coli* Nissle 1917 wild type and mutant strains (see Tab. 1) for 3 hours. Treatment with EcN wt, EcNΔ*csgBA* (curli-negative), EcNΔ*fim* (Type 1 pili) and EcNΔ*foc* (F1C pili) led to a significant downregulation of Hes1 (A) and Hath1 (B) transcripts, whereas HBD2 (C) and Muc1 (D) mRNA was upregulated. In contrast, EcNΔ*fliA* (sigma factor of flagellin), EcNΔ*fliC* (flagellin), EcNΔ*flgE* (hook) lost the regulation ability. Data represent the means ± SEM normalised to basal expression of untreated controls set at 1 (n = 3). *: p<0.05.

Consistent with our previous observations, KLF4 (Fig. S5A) and Muc2 (Fig. S5B) mRNA levels were unchanged following a 3 hour treatment with *E. coli* Nissle 1917 wild type and mutant strains. In contrast, Hes1 (0.61-fold, p = 0.046, Fig. 5A) and Hath1 (0.67-fold, p = 0.035, Fig. 5B) mRNA levels were significantly downregulated after 3 hours of treatment with wild type *E. coli* Nissle 1917 as compared to untreated controls, whereas HBD2 (1246-fold, p = 0.005, Fig. 5C) and Muc1 (3.1-fold, p = 0.014, Fig. 5D) mRNA transcripts were upregulated. This change in Hes1 and Hath1 expression pattern was similar after incubation with the *E. coli* Nissle 1917 mutant strains EcNΔ*csgBA* (Hes1∶0.45-fold, p = 0.015, Hath1∶0.70-fold, p = 0.044, HBD2∶1775-fold, p = 0.002, Muc1∶2.5-fold, p = 0.034), EcNΔ*fim* (Hes1∶0.51-fold, p = 0.038, Hath1∶0.60-fold, p = 0.016, HBD2∶1684-fold, p = 0.001, Muc1∶2.3-fold, p = 0.078) and EcNΔ*foc* (Hes1∶0.56-fold, p = 0.067, Hath1∶0.72-fold, p = 0.018, HBD2∶1441-fold, p = 0.002 Muc1∶2.6-fold, p = 0.016). In contrast, incubation with the flagellin mutants EcNΔ*fliA*, EcNΔ*fliC* and EcNΔ*flgE* left Hes1, Hath1, HBD2 and Muc1 mRNA transcripts unchanged as compared to the untreated controls (Fig. 5A–D). These results clearly demonstrate that the flagellin of *E. coli* Nissle 1917 is essential for regulating Hes1, Hath1, HBD2 and Muc1 mRNA expression.

### Microbiota also Regulate Hes1, Math1 and KLF4 *in vivo*

**Figure 6:**
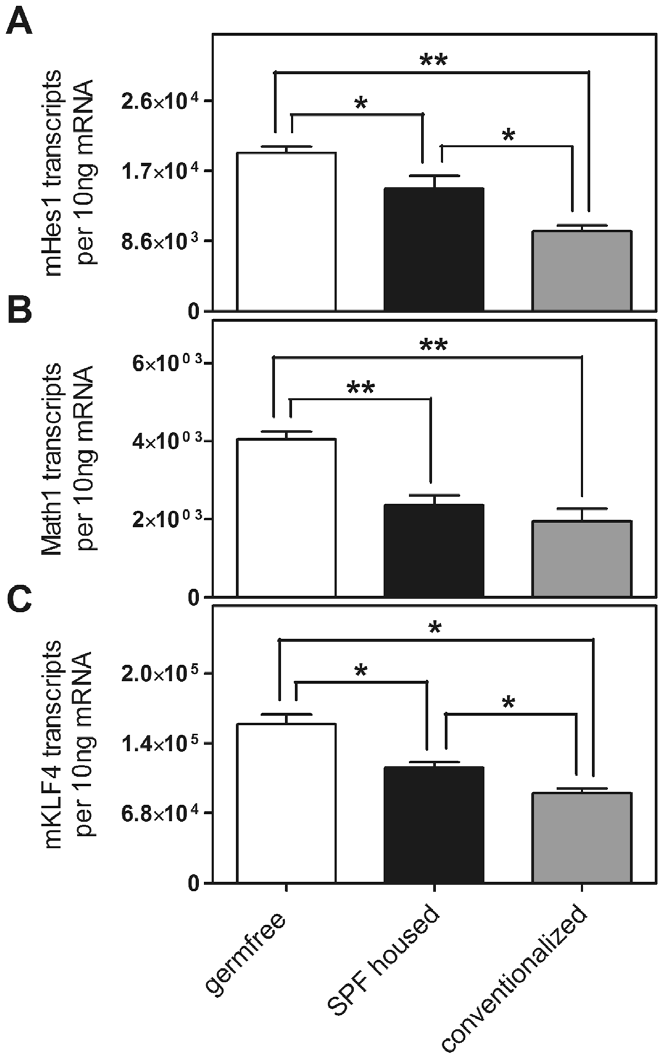
Mouse (m) Hes1, Math1 and mKLF4 mRNA expression in colon of germ free (n = 7), SPF (specific pathogen free, n = 4) and conventionalized mice (n = 4). The presence of intestinal microbiota is associated with downregulation of mHes1 (A), Math1 (B) and mKLF4 (C) mRNA in SPF mice and even more in conventionalized mice. Data represent the means ± SEM normalised to basal expression of untreated controls set at 1. *: p<0.05, **: p<0.01.

As our *in vitro* observations suggested an effect of bacteria on intestinal epithelial cell differentiation, we next evaluated the *in vivo* relevance of our findings by using germfree animals to assess the role of the intestinal microflora in the regulation of epithelial cell differentiation in mice. Therefore, mRNA expression of mHes1, Math1 (the mouse homolog of Hath1), mKLF4, mMuc1 and mMuc2 was analyzed in the colon of germfree mice as compared to mice reared with specific pathogen free (SPF) intestinal microflora (m…mouse). In the colon of SPF mice, mRNA expression of mHes1 (0.77-fold, p = 0.04), Math1 (0.58-fold, p = 0.006) and mKLF4 (0.73-fold, p = 0.011) was lower in comparison to germfree mice (Fig. 6A–C). These results are consistent with our *in vitro* observations suggesting an inhibitory effect of bacteria on expression of these differentiation genes. In order to further evaluate this, we also analyzed colons from germfree mice that regained their microbiota by cohousing with SPF mice for 4 weeks. These conventionalised mice showed even more decreased mRNA levels for mHes1 (0.51-fold p = 0.006), Math1 (0.48-fold, p = 0.006) and mKLF4 (0.57-fold p = 0.011) as compared to the germfree mice (Fig. 6A–C), again confirming the inhibitory effect of the microbiota on expression of these differentiation factors. Surprisingly however, this microbiota effect was not reflected in the expression levels of mMuc1 (Fig. S6A) and mMuc2 (Fig. S6B) since they were not significantly altered in germfree versus colonized mice.

**Figure 7:**
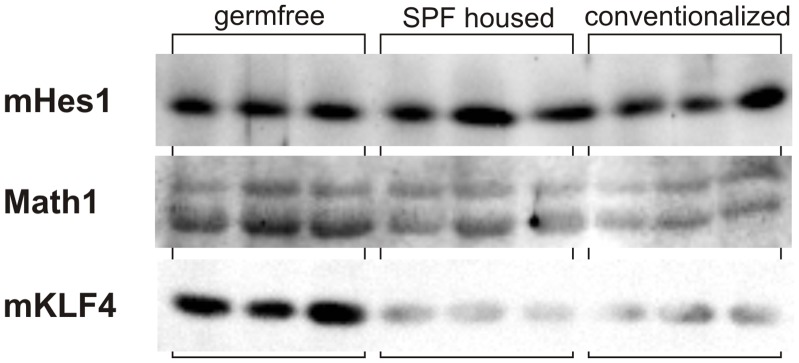
Mouse (m) Hes1, Math1 and mKLF4 protein expression in colon of germ free, SPF (specific pathogen free) and conventionalized mice. The presence of intestinal microbiota is associated with a downregulation of Math1 and mKLF4 but not mHes1 protein in SPF and conventionalized mice.

**Figure 8:**
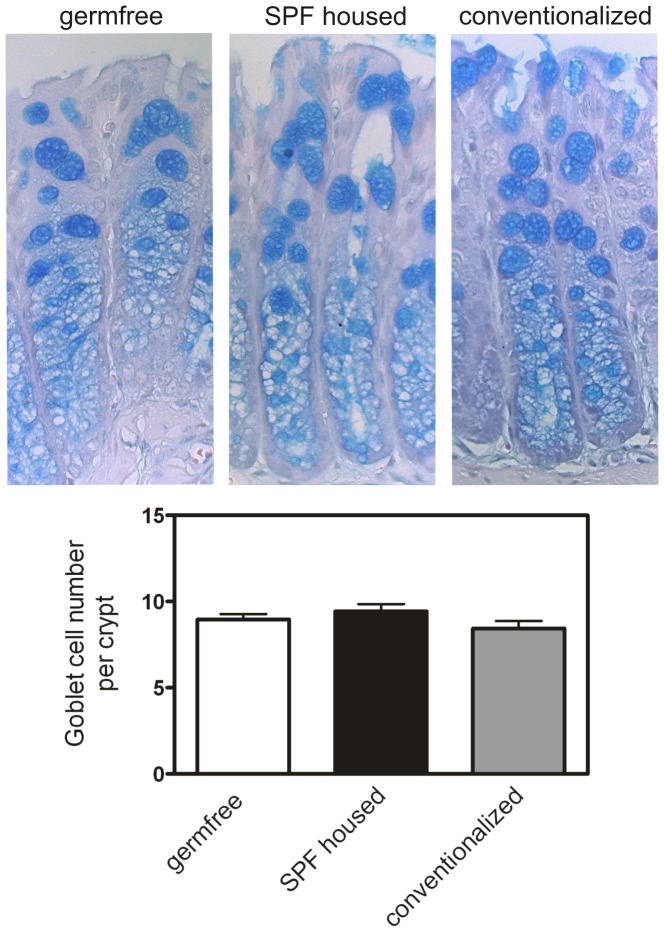
Goblet cell number in colon of germ free, SPF (specific pathogen free) and conventionalized mice. The number of goblet cells is unchanged between the three subgroups.

In accordance with the mRNA data we also found a diminished colonic Math1 and mKLF4 protein expression by Western blot analysis in SPF housed and conventionalized mice as compared to the germfree animals (Fig. 7). In contrast, mHes1 protein seems to be unchanged in these three groups (Fig. 7). However, the diminished expression of the goblet cell differentiation markers Math1 and mKLF4 does not lead to a reduced number of goblet cells as shown in Fig. 8.

## Discussion

The current study focused on the regulatory effects of intestinal bacteria on the expression of the intestinal epithelial differentiation factors Hes1, Hath1 and KLF4. Moreover the bacterial effects on mucins Muc1 and Muc2, as well as the defensin HBD2 were investigated as well.

We found a bacterial regulation of the transcription factors Hes1, Hath1 and KLF4 in the colon adenocarcinoma cell line LS174T, especially by *E. coli* K-12 and *E. coli* Nissle 1917. These changes in mRNA expression were confirmed for Hes1 and Hath1 (and also in trend for KLF4) on the protein level by Western blot experiments following stimulation with *E. coli* Nissle 1917. Notably, in case of Hes1, a higher molecular weight band appeared following treatment with *E. coli* Nissle 1917. This double band did not occur in the mouse colon where only a single band was observed, therefore we conclude that the double band is an *in vitro* phenomenon of cell culture and has likely no physiological relevance *in vivo.* However, to our knowledge, this is the first study that shows bacteria to regulate these three epithelial differentiation factors in colonic epithelial cells *in vitro*. Prior observations demonstrated Hes1 to be induced by *Porphyromonas gingivalis* lipopolysaccharides in the mouse osteoblastic cell line MC3T3E-1 and in primary mouse bone marrow stromal cells [35]. Moreover *Mycobacterium bovis* led to increased Hes1 transcripts in peritoneal mice macrophages [36] whereas *Salmonella typhimurium* causes a decrease of Hes1 expression in PS cells [37]. In case of Hath1, there are no data available concerning the interaction with bacteria at all, whereas KLF4 was shown to be induced in macrophages again by *P. gingivalis* lipopolysaccharides [38]. In the present study Muc1 expression of LS174T cells was also significantly induced by several bacteria, such as *E. coli* Nissle 1917 and *E. coli* K-12, whereas Muc2 expression was unchanged following incubation with all bacteria strains tested. Accordingly, immunostaining on LS174T cells showed a clear induction of Muc1 protein following stimulation with *E. coli* Nissle 1917 as compared to untreated cells, whereas Muc2 protein was unaffected. Several, in part conflicting studies, focused on the impact of bacteria on the expression profiles of the mucins Muc1 and Muc2 in intestinal epithelial cells. For instance, HT29 cells treated with *E. coli* Nissle 1917 did not alter the mRNA and protein expression of these two mucins. In contrast, the probiotic cocktail VSL#3 induced Muc2 secretion in HT29 cells [39] but not in LS174T cells [40]. Moreover, *L. acidophilus* enhanced Muc2 transcripts in HT29 cells [41], whereas another group could not confirm these data [42]. A recent study showed a strong up-regulation of Muc2 in LS174T cells after treatment with flagellin from *Salmonella typhimurium* [43]. Differences between these prior observations and our data could be explained by the use of different cell lines (e.g. HT-29 vs. LS174T cells) and possibly also by different bacterial preparations (e.g. living vs. heat-inactivated bacteria).

In addition, we found HBD2 transcripts to be upregulated by *E. coli* Nissle 1917, *E. coli* K-12, and other bacteria. This is in principle consistent with prior data where HBD2 mRNA was demonstrated to be induced in Caco-2 cells following a treatment with *E. coli* Nissle 1917, uropathogenic *E. coli*, as well as *L. fermentum, L. acidophilus* and VSL#3, but not with *E. coli* K-12 [30]. Moreover, in LS174T cells the expression of HBD2 was shown to be elevated once treated with *E. coli* D21, *Micrococcus luteus* and *Salmonella typhimurium* [44]. Notably, the effect of *L. fermentum* on HBD2 expression was clearly higher in Caco-2 than in LS174T cells. This also implies that HBD2 regulation varies in different cell lines.

Overall, cell culture experiments showed a stronger downregulation of the columnar cell differentiation marker Hes1, as compared to the secretory cell differentiation marker Hath1. Therefore, it could be speculated that specific bacteria such as *E. coli* Nissle 1917 and *E. coli* K-12 may influence the differentiation of specific cell lineages with a shift towards the goblet cell lineage. Nevertheless, the interplay of underlying mechanisms and the exact consequences of the effects on the differentiation markers need further study.

Previously, the induction of HBD2 by *E. coli* Nissle 1917 was demonstrated to be dependent on flagellin [34]. Since Hes1, Hath1 and Muc1 were also regulated by *E. coli* Nissle 1917, we analyzed the role of flagellin with respect to these three factors. In contrast to *E. coli* Nissle 1917 wild type, Hes1 and Hath1 mRNA was not downregulated by the flagellin mutant strains EcNΔ*fliA*, EcNΔ*fliC* and EcNΔ*flgE*. Accordingly, Muc1 expression was enhanced in *E. coli* Nissle 1917 wild type, but not in EcNΔ*fliA*, EcNΔ*fliC* and EcNΔ*flgE*. This implies that Hes1, Hath1 and Muc1 are regulated by *E. coli* Nissle 1917 flagellin, similar to HBD2.

To elucidate the effect of the intestinal microflora *in vivo,* we analysed the expression of mHes1, Math1 and mKLF4 in the colon of germ free mice compared to SPF and conventionalized mice. Similar to the cell culture data, we observed a significantly lower Math1 and mKLF4 mRNA and protein expression in colonized mice compared to germ free mice, whereas mHes1 expression was reduced on mRNA but not on protein level. This difference in mHes1 expression could be a result of posttranscriptional regulation mechanisms which need further investigations.

Several arguments underline that intestinal bacteria play a crucial role in IBD pathogenesis: Inflammation in IBD is located in areas with a high density of bacteria (mostly colon and/or terminal ileum) [45]; germ free mice do not develop colitis [46]; exposure of fecal stream to the terminal ileum worsen inflammation [47]; antimicrobial peptides are insufficiently expressed in CD, and mutations of human receptors recognizing luminal bacteria, such as NOD2 [48],[49] and TLR dysfunction [50],[51] are linked to a higher risk of IBD development. Moreover, the intestinal microflora is altered in IBD as compared to healthy controls. Numerous studies described changes in the composition of the microflora between CD, UC and healthy patients [52]–[54], and mucosa-associated and even intracellular bacteria were found in both types of IBD [17], [18]. Recent studies showed UC to be associated with goblet cell [21] and ileal CD with Paneth cell differentiation defects [20]. In addition, mice with an epithelial-specific defect leading to reduced Hes1 expression were recently shown to spontaneously develop colitis [55]. Considering these observations, our data suggest that in addition to the genetic predisposition, the luminal microbiota may directly affect epithelial differentiation and its defensive role.

There are also reasons to suggest bacteria to be involved in colon cancer pathogenesis: intestinal cancer is mostly found in the colon, the segment with the highest number of bacteria [56], some bacteria can induce malignancies, e.g. H. pylori and gastric neoplasia [57],[58], and, moreover, patients with colon cancer have adherent bacteria [27] as well as more circulating antibodies against specific bacteria (e.g. S. gallolyticus) compared to healthy controls [59]. On the other hand, several studies reported that probiotics, such as *L. acidophilus* NCFM, suppress carcinogenesis [60], [61]. In most colorectal cancers Notch signaling was found to be activated [62], [63], whereas Hath1 and KLF4 were decreased [64]–[67]. It may be speculated that the downregulation of the important epithelial cell differentiation factors Hath1 and KLF4 could play a role in this regard.

Taken together, the current study shows that intestinal bacteria regulate the epithelial differentiation factors Hes1, Hath1 and KLF4 *in vitro* and *in vivo*. This could be involved in IBD and colon cancer pathogenesis although further details remain to be elucidated.

## Supporting Information

Figure S1 **Hes1, Hath1 and KLF4 mRNA expression in LS174T cells after treatment with different heat-inactivated bacteria for 12 hours.** Hes1 expression was diminished by Symbioflor G2, *E. coli* K-12, *E. coli* Nissle 1917 and *L. acidophilus* (A). Hath1 transcripts were downregulated by *E. coli* K-12 and *E. coli* Nissle 1917 (B). KLF4 mRNA was impaired by *E. coli* K-12, *E. coli* Nissle 1917, *L. acidophilus* and *B. vulgatus* (C). Data represent the means ± SEM normalised to basal expression of untreated controls set at 1 (n = 4). *: p<0.05, **: p<0.01, ***: p<0.001.
(TIF)

Figure S2 **HBD2, Muc1 and Muc2 mRNA expression in LS174T cells after treatment with different heat-inactivated bacteria for 12 hours.** HBD2 expression was induced by Symbioflor G2, *E. coli* K-12, *E. coli* Nissle 1917 and *B. breve* (A). Muc1 transcripts were upregulated by Symbioflor G2, *E. coli* K-12 and *E. coli* Nissle 1917 (B). Muc2 mRNA was unchanged (C). Data represent the means ± SEM normalised to basal expression of untreated controls set at 1 (n = 4). *: p<0.05, **: p<0.01, ***: p<0.001.
(TIF)

Figure S3 **Hes1, Hath1 and KLF4 mRNA expression in LS174T cells following treatment with** ***E. coli*** **Nissle 1917, DBZ and** ***E. coli*** **Nissle 1917+ DBZ for 3, 6, 12 and 24 hours.** DBZ led to a strong downregulation of Hes1 after 3 to 24 hours treatment (A), a significant upregulation of Hath1 after 6 to 24 hours treatment (B) and an increase of KLF4 mRNA expression following 24 hours treatment (C). Data represent the means ± SEM normalised to basal expression of untreated controls set at 1 (n = 4). *: p<0.05, **: p<0.01, ***: p<0.001.
(TIF)

Figure S4 **HBD2, Muc1 and Muc2 mRNA expression in LS174T cells following treatment with** ***E. coli*** **Nissle 1917, DBZ and** ***E. coli*** **Nissle 1917+ DBZ for 3, 6, 12 and 24 hours.** *E. coli* Nissle 1917 upregulated HBD2 and Muc1 transcripts independent of DBZ treatment (A+B). Muc2 mRNA expression was unchanged by DBZ and/or *E. coli* Nissle 1917 (C). Data represent the means ± SEM normalised to basal expression of untreated controls set at 1 (n = 4). *: p<0.05, **: p<0.01, ***: p<0.001.
(TIF)

Figure S5 **KLF4 and Muc2 mRNA expression in LS174T cells incubated with heat inactivated** ***E. coli*** **Nissle 1917 wild type and mutant strains (see** **Tab. 1** **) for 3 hours.** KLF4 (A) and Muc2 (B) mRNA expression was unchanged in *E. coli* Nissle wild type (EcN wt) and mutant strains (EcNΔ*fliA*, EcNΔ*fliC*, EcNΔ*flgE*, EcNΔ*csgBA*, EcNΔ*fim*, EcNΔ*foc*). Data represent the means ± SEM normalised to basal expression of untreated controls set at 1 (n = 4).
(TIF)

Figure S6 **Mouse (m) Muc1 and Muc2 mRNA expression in colon of germ free (n = 7), SPF (specific pathogen free, n = 4) and conventionalized mice (n = 4).** No significant changes on mMuc1 (A) and mMuc2 (B) expression were found between the subgroups.
(TIF)
