# Correction: Bacteria Regulate Intestinal Epithelial Cell Differentiation Factors Both *In Vitro* and *In Vivo*

**DOI:** 10.1371/annotation/13f3d7ee-a72c-4879-bedb-8e73b6b364ac

**Published:** 2013-05-09

**Authors:** Svetlana Becker, Tobias A. Oelschlaeger, Andy Wullaert, Katerina Vlantis, Manolis Pasparakis, Jan Wehkamp, Eduard F. Stange, Michael Gersemann

Katerina Vlantis should have been included as the fourth author in the byline.

Her affiliation is: 4 Institute for Genetics, University of Cologne, Cologne, Germany

The correct citation is: Becker S, Oelschlaeger TA, Wullaert A, Vlantis K, Pasparakis M, et al. (2013) Bacteria Regulate Intestinal Epithelial Cell Differentiation Factors Both *In Vitro* and *In Vivo*. PLoS ONE 8(2): e55620. doi:10.1371/journal.pone.0055620 

