# Supplementary figures and images for: Bacteria Regulate Intestinal Epithelial Cell Differentiation Factors Both In Vitro and In Vivo
Source: PLoS One. 2013 Feb 13;8(2):e55620. doi: 10.1371/journal.pone.0055620 (PMC3572096; doi:10.1371/journal.pone.0055620)

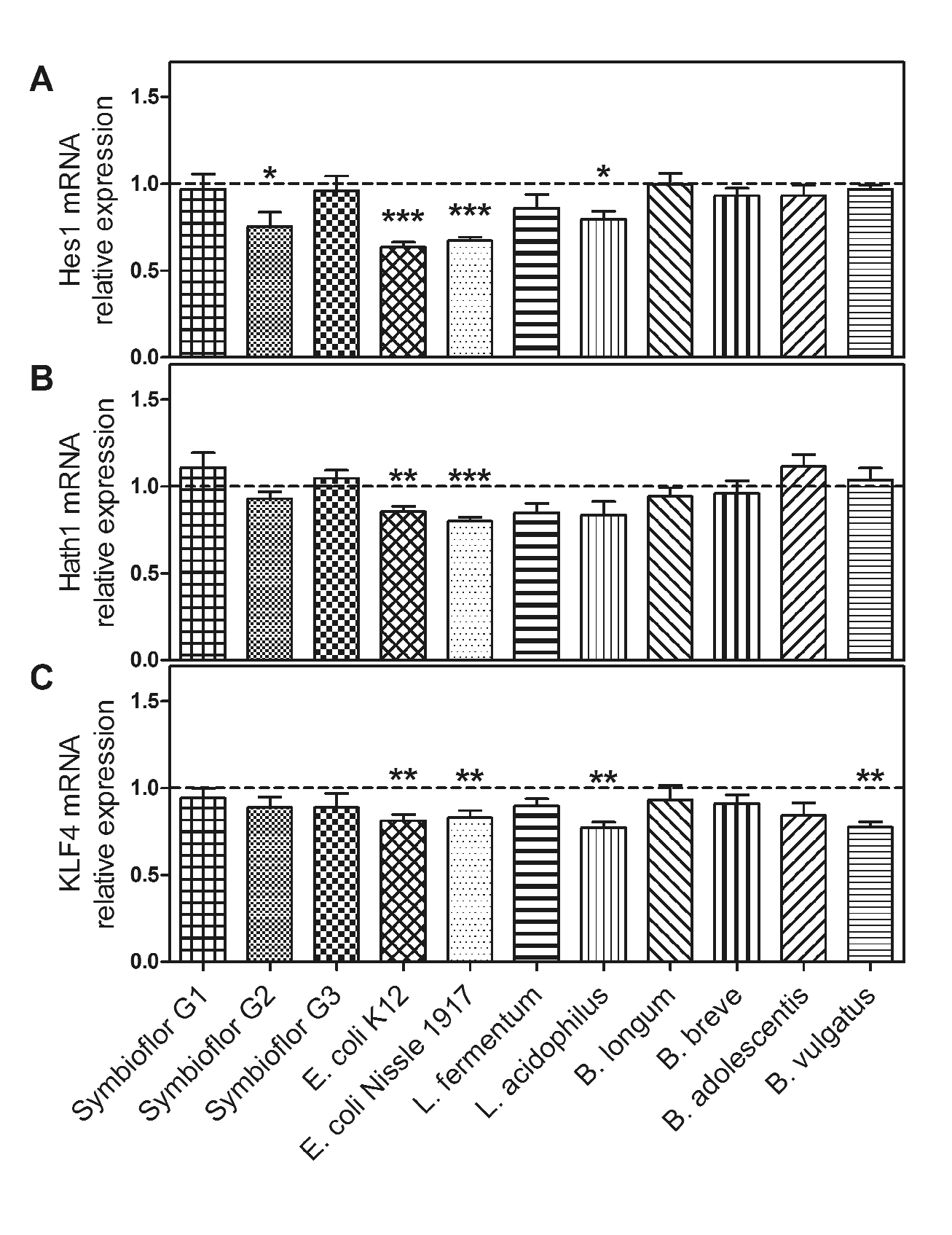

Supplement: Figure S1 — Hes1, Hath1 and KLF4 mRNA expression in LS174T cells after treatment with different heat-inactivated bacteria for 12 hours. Hes1 expression was diminished by Symbioflor G2, E. coli K-12, E. coli Nissle 1917 and L. acidophilus (A). Hath1 transcripts were downregulated by E. coli K-12 and E. coli Nissle 1917 (B). KLF4 mRNA was impaired by E. coli K-12, E. coli Nissle 1917, L. acidophilus and B. vulgatus (C). Data represent the means ± SEM normalised to basal expression of untreated controls set at 1 (n = 4). *: p<0.05, **: p<0.01, ***: p<0.001. (TIF) [file pone.0055620.s001.tif]

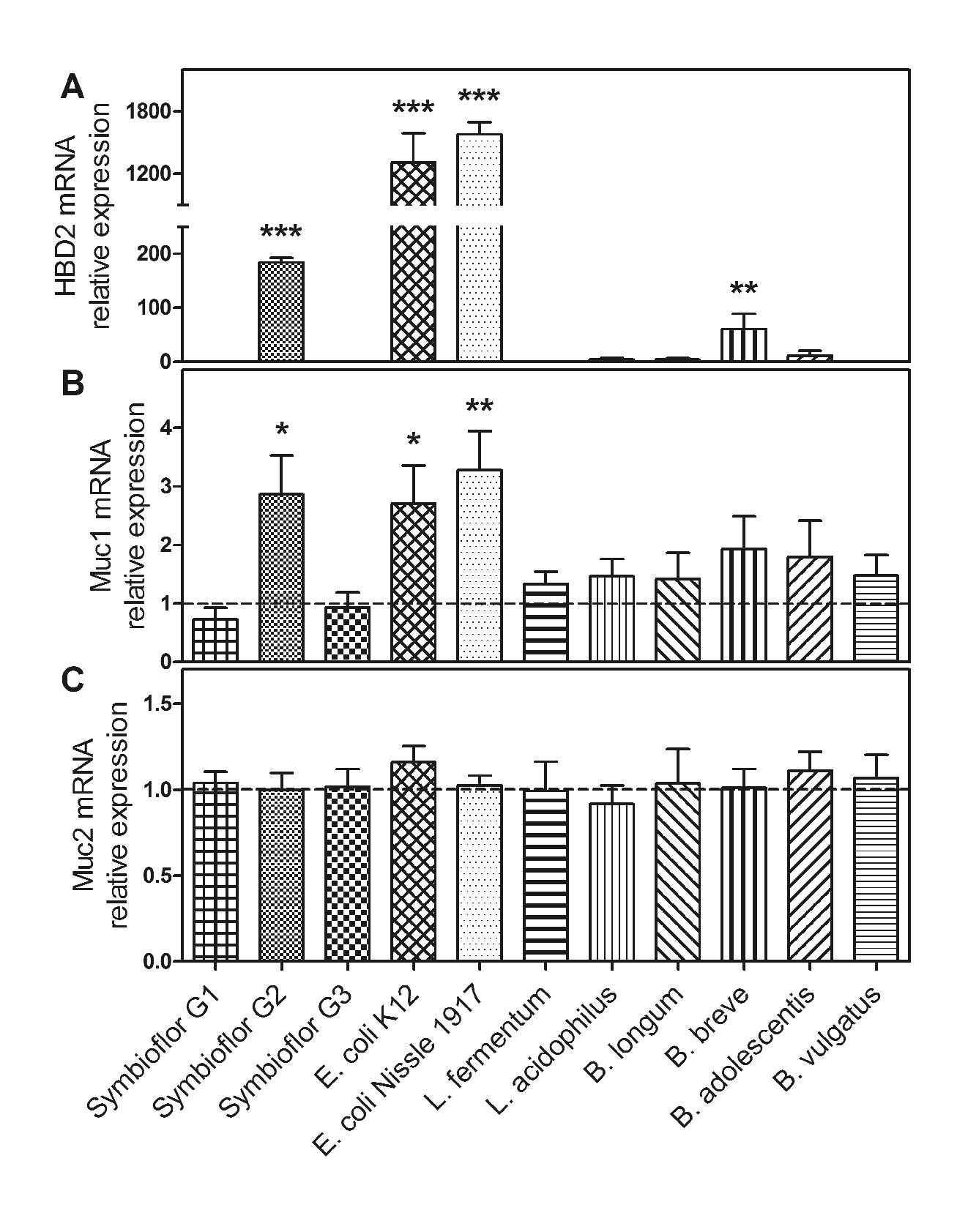

Supplement: Figure S2 — HBD2, Muc1 and Muc2 mRNA expression in LS174T cells after treatment with different heat-inactivated bacteria for 12 hours. HBD2 expression was induced by Symbioflor G2, E. coli K-12, E. coli Nissle 1917 and B. breve (A). Muc1 transcripts were upregulated by Symbioflor G2, E. coli K-12 and E. coli Nissle 1917 (B). Muc2 mRNA was unchanged (C). Data represent the means ± SEM normalised to basal expression of untreated controls set at 1 (n = 4). *: p<0.05, **: p<0.01, ***: p<0.001. (TIF) [file pone.0055620.s002.tif]

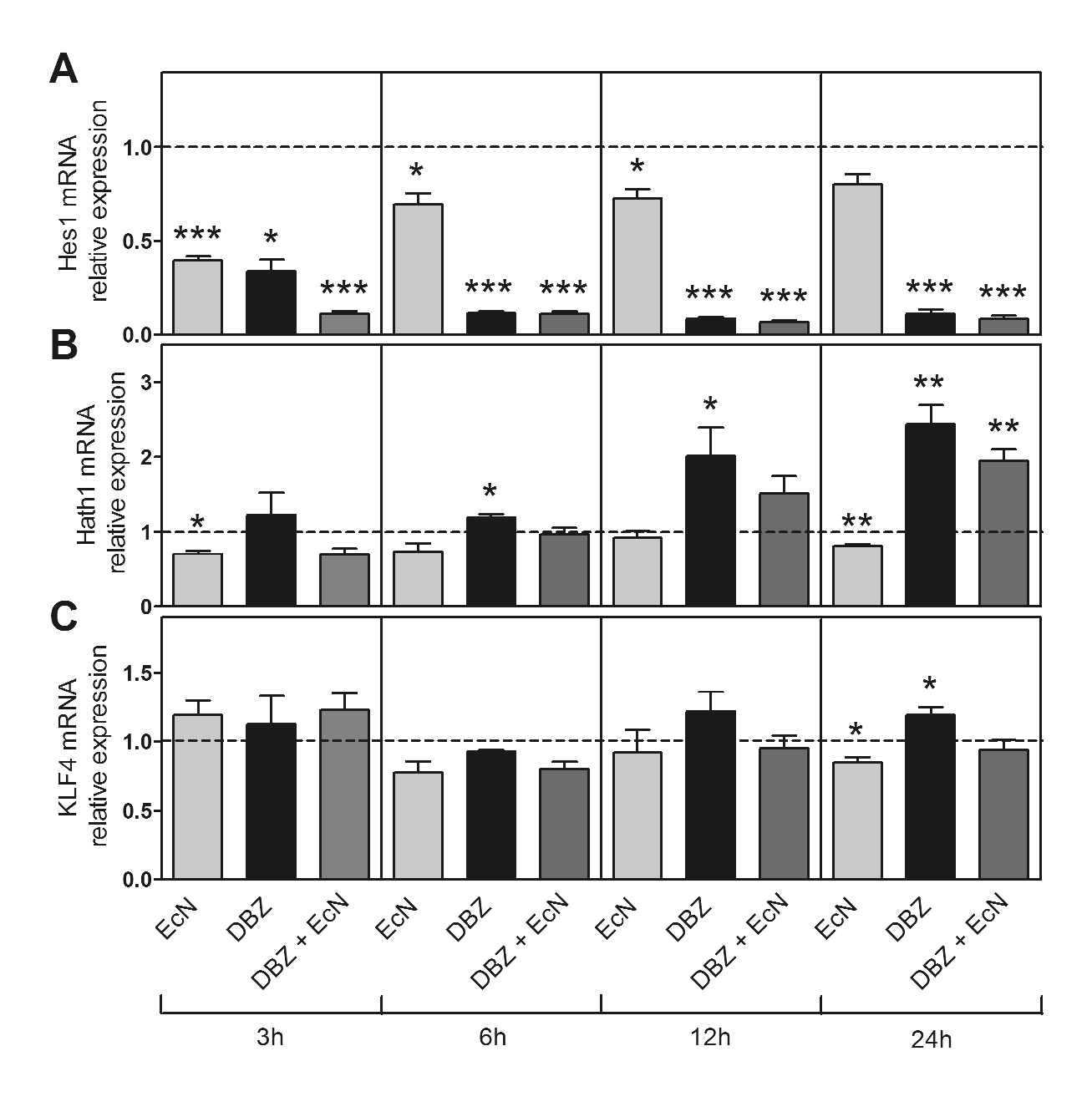

Supplement: Figure S3 — Hes1, Hath1 and KLF4 mRNA expression in LS174T cells following treatment with E. coli Nissle 1917, DBZ and E. coli Nissle 1917+ DBZ for 3, 6, 12 and 24 hours. DBZ led to a strong downregulation of Hes1 after 3 to 24 hours treatment (A), a significant upregulation of Hath1 after 6 to 24 hours treatment (B) and an increase of KLF4 mRNA expression following 24 hours treatment (C). Data represent the means ± SEM normalised to basal expression of untreated controls set at 1 (n = 4). *: p<0.05, **: p<0.01, ***: p<0.001. (TIF) [file pone.0055620.s003.tif]

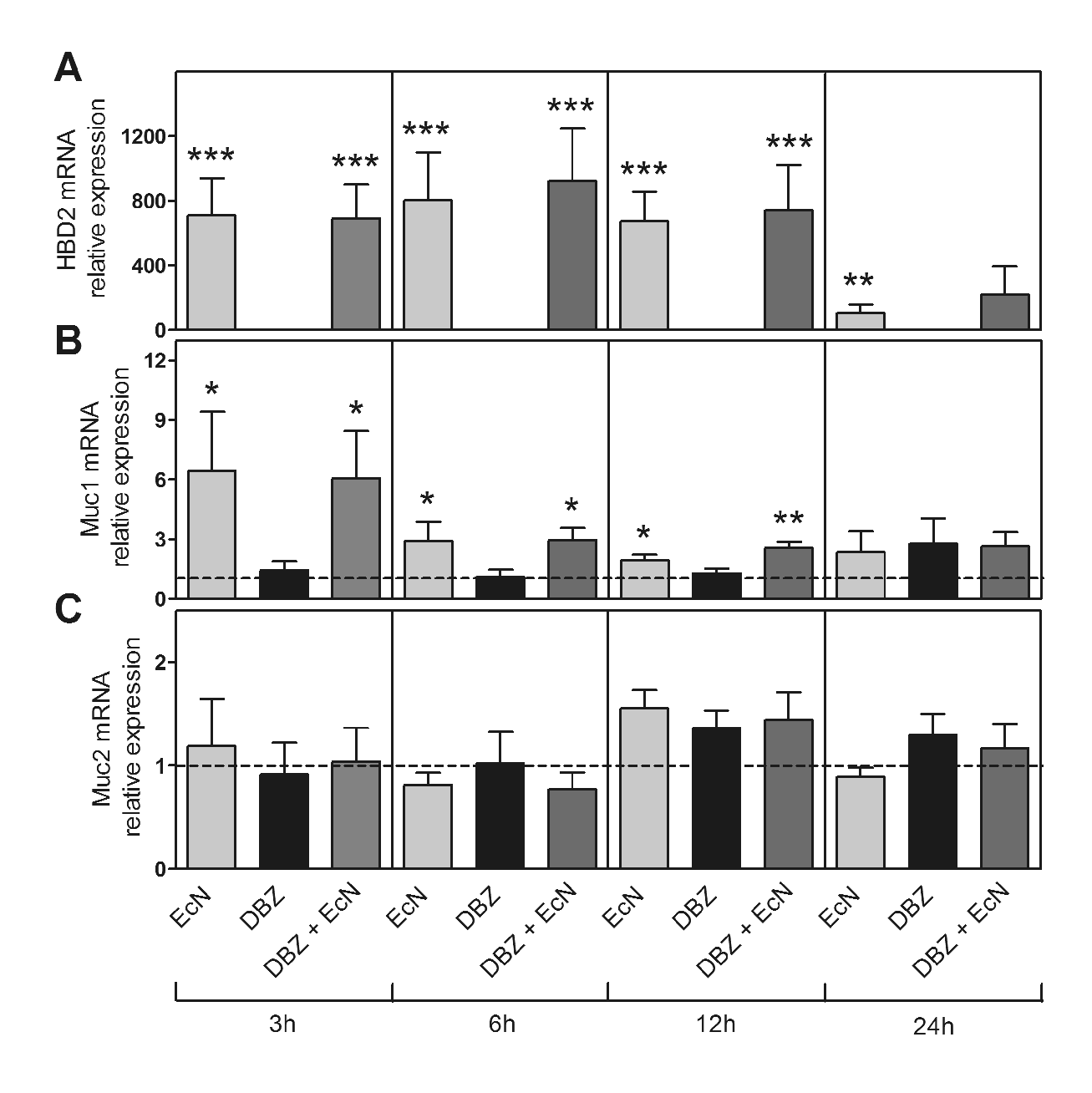

Supplement: Figure S4 — HBD2, Muc1 and Muc2 mRNA expression in LS174T cells following treatment with E. coli Nissle 1917, DBZ and E. coli Nissle 1917+ DBZ for 3, 6, 12 and 24 hours. E. coli Nissle 1917 upregulated HBD2 and Muc1 transcripts independent of DBZ treatment (A+B). Muc2 mRNA expression was unchanged by DBZ and/or E. coli Nissle 1917 (C). Data represent the means ± SEM normalised to basal expression of untreated controls set at 1 (n = 4). *: p<0.05, **: p<0.01, ***: p<0.001. (TIF) [file pone.0055620.s004.tif]

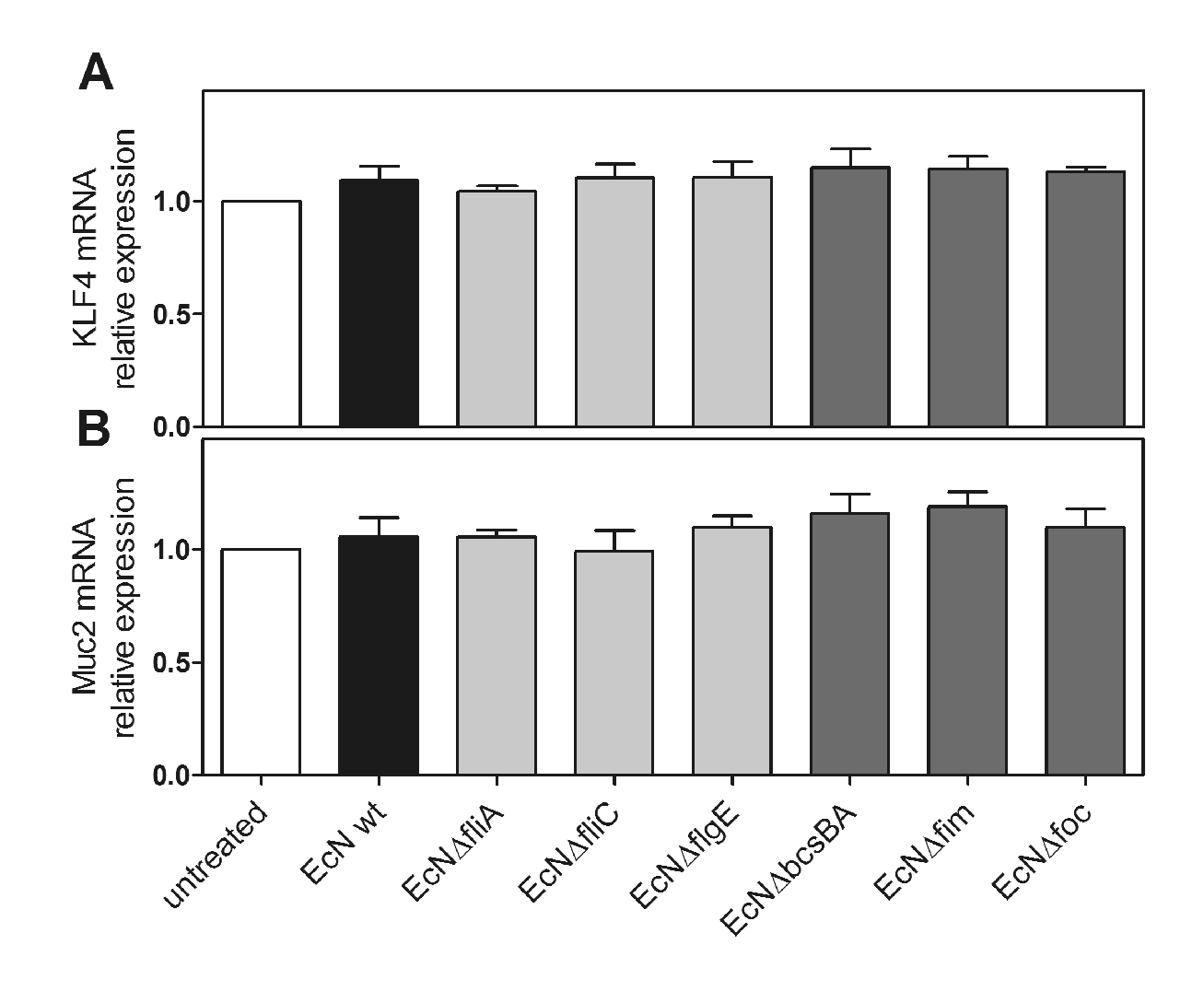

Supplement: Figure S5 — KLF4 and Muc2 mRNA expression in LS174T cells incubated with heat inactivated E. coli Nissle 1917 wild type and mutant strains (see Tab. 1 ) for 3 hours. KLF4 (A) and Muc2 (B) mRNA expression was unchanged in E. coli Nissle wild type (EcN wt) and mutant strains (EcNΔfliA, EcNΔfliC, EcNΔflgE, EcNΔcsgBA, EcNΔfim, EcNΔfoc). Data represent the means ± SEM normalised to basal expression of untreated controls set at 1 (n = 4). (TIF) [file pone.0055620.s005.tif]

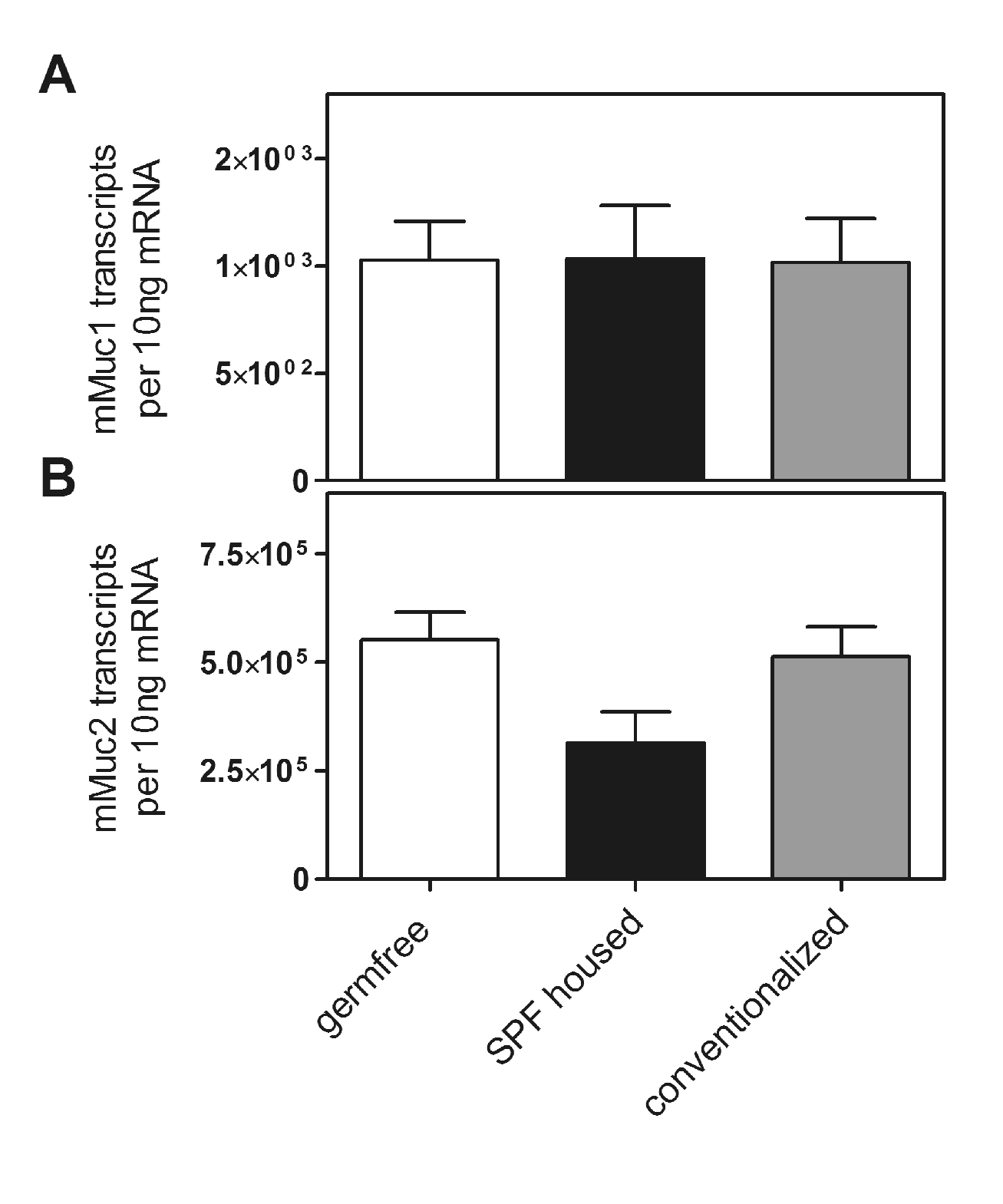

Supplement: Figure S6 — Mouse (m) Muc1 and Muc2 mRNA expression in colon of germ free (n = 7), SPF (specific pathogen free, n = 4) and conventionalized mice (n = 4). No significant changes on mMuc1 (A) and mMuc2 (B) expression were found between the subgroups. (TIF) [file pone.0055620.s006.tif]
